# Population structure and acquisition of the *vanB* resistance determinant in German clinical isolates of *Enterococcus faecium* ST192

**DOI:** 10.1038/srep21847

**Published:** 2016-02-23

**Authors:** Jennifer K. Bender, Alexander Kalmbach, Carola Fleige, Ingo Klare, Stephan Fuchs, Guido Werner

**Affiliations:** 1 National Reference Centre for Staphylococci and Enterococci, Robert Koch Institute, Department of Infectious Diseases, Wernigerode, 38855, Germany

## Abstract

In the context of the global action plan to reduce the dissemination of antibiotic resistances it is of utmost importance to understand the population structure of resistant endemic bacterial lineages and to elucidate how bacteria acquire certain resistance determinants. Vancomycin resistant enterococci represent one such example of a prominent nosocomial pathogen on which nation-wide population analyses on prevalent lineages are scarce and data on how the bacteria acquire resistance, especially of the *vanB* genotype, are still under debate. With respect to Germany, an increased prevalence of VRE was noted in recent years. Here, invasive infections caused by sequence type ST192 VRE are often associated with the *vanB*-type resistance determinant. Hence, we analyzed 49 *vanB*-positive and *vanB*-negative *E. faecium* isolates by means of whole genome sequencing. Our studies revealed a distinct population structure and that spread of the Tn*1549*-*vanB*-type resistance involves exchange of large chromosomal fragments between *vanB*-positive and *vanB*-negative enterococci rather than independent acquisition events. *In vitro* filter-mating experiments support the hypothesis and suggest the presence of certain target sequences as a limiting factor for dissemination of the *vanB* element. Thus, the present study provides a better understanding of how enterococci emerge into successful multidrug-resistant nosocomial pathogens.

Regarded as a gut commensal organism of animals and humans alike, with the potential to incidentally invade underlying tissue and being capable to withstand harsh environmental condition, enterococci have long been recognized as an important nosocomial pathogen. Life-threatening diseases such as endocarditis or sepsis can be elicited mostly in immunocompromised patients, elderly or neonates. Due to their often multi-drug resistant phenotype only a few therapeutic options are left to treat infections caused by vancomycin-resistant enterococci (VRE). Although VRE are of generally low prevalence in European clinics compared to U.S. hospitals^1^,^2^, surveillance data from the European Antimicrobial Resistance Surveillance Network (EARS-Net) clearly demonstrate that VRE are on the rise in European countries (http://ecdc.europa.eu). please change to: It was observed a steady increase of VRE in Germany in recent years. As for instance 14.5% of all invasive *E. faecium* isolates were tested vancomycin-resistant in 2013.

Vancomycin resistance is mediated through expression of a gene cluster of which nine different genotypes, VanA to VanN, can be distinguished on the basis of the central ligase^3^. VanA and VanB account for the majority of clinically important VR-*E. faecalis* and VR-*E. faecium* cases and are usually associated with mobile genetic elements (MGE) such as transposons^4^,^5^. While *vanA* is dominant in the USA and in most of Europe^6^, the National Reference Centre for Staphylococci and Enterococci in Germany has recognized a steady increase of *vanB*-type VRE since 2010 (EARS-Net annual report 2013: http://ecdc.europa.eu/en/publications/_layouts/forms/Publication_DispForm.aspx?List=4f55ad51-4aed-4d32-b960-af70113dbb90&ID=1205#sthash.M5euPg56.dpuf). We recently reported about the ongoing high prevalence of *vanB*-type *E. faecium* of sequence type (ST) 192 on a neonatal care unit^7^. As recognized by the German NRC between 2011 and 2014, ST192 (33%) represents the most prevalent ST besides ST117 (25%) and ST17 (16%) isolated from bloodstream infections and is primarily associated with the *vanB* genotype (NRC annual reports).

The *vanB* locus is mainly encoded by a conjugative transposon of the Tn*1549*-/Tn*5382*-subtype and of chromosomal origin. However, localization on pRUM-like plasmids has also been reported from Sweden, Spain or Singapore^8^,^9^. It is still under debate how the *vanB* transposon facilitates its own transfer, as detailed investigations yielded conflicting results regarding the mechanism of transmission. Launay and co-workers demonstrated that Tn*1549* represents a genuine transposon capable of forming a circular intermediate in order to be transferred and integrated into the target bacterial chromosome in the gut of gnotobiotic mice^10^. Contradicting these results, *in vitro* experiments performed by Quintiliani, Carias, and others show the movement of large chromosomal elements which would favor a mechanism related to homologous or illegitimate recombination^11^,^12^,^13^,^14^. Interestingly, Tn*1549-vanB* is not restricted to enterococci but was also detected among anaerobic bacilli such as *Clostridium* spp. or *Eggerthella lenta*^10^,^15^,^16^,^17^,^18^. It has been hypothesized that the myriad of reservoirs constitute a source for frequent *vanB* transposition events and thus acquisition of resistance from gut commensals by susceptible *Enterococcus* recipients^19^.

As *vanB*-positive *E. faecium* ST192 led to a marked increase of VRE outbreaks and became a highly prevalent lineage of bloodstream infections in Germany in recent years, the present study was conducted to investigate VRE of this particular sequence type with respect to i) population structure, ii) acquisition of the *vanB* resistance determinant and iii) transfer of the resistance locus.

## Results

### Description of isolates

The vast majority of the 49 *E. faecium* strains used in this study was isolated from invasive infections between 2004 and 2014 and sent to our National Reference Centre for Staphylococci and Enterococci from hospitals all across Germany (Table 1). As strains of ST192 have been most prevalent in recent years and have been associated with hospital-related outbreaks in German clinics^7^, we set out to analyze *E. faecium* ST192 in more detail with respect to strain relatedness and acquisition of the composite *vanB* transposon Tn*1549*. Thus, 39 of the 49 isolates from this study belonged to ST192 whilst the remaining strains represent further prevalent German STs such as ST117, ST17, ST78 or ST203 and were included for comparative reasons. First, strains were analyzed phenotypically, hence displaying variable levels of vancomycin MICs from as low as 2 μg/ml up to 512 μg/ml for *vanB*-positive isolates, respectively (Table 1). As expected, *vanB*-negative strains were fully susceptible to vancomycin (≤1 μg/ml) with one exception of UW11625 which harbored the *vanA* gene cluster but was negative in *vanB*-specific PCR experiments (data not shown).

### Population structure of ST192 and non-ST192 *E. faecium* in Germany

**Figure 1:**
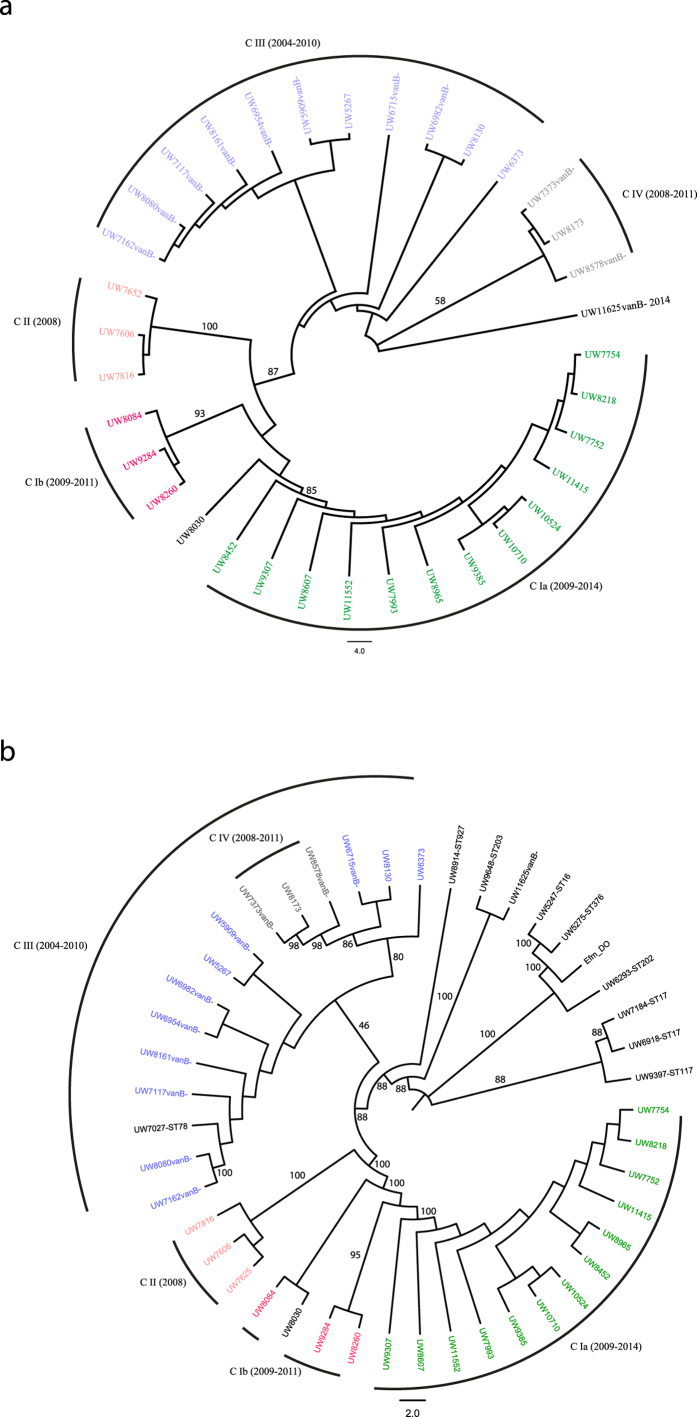
Phylogenetic analysis of the core genome of German *E. faecium* ST192 (**a**) and non-ST192 (**b**) clinical isolates. (**a**) The proportional transformed branch diagram revealed 4 different clades or subclades of ST192 isolates. (**b**) ST192 and non-ST192 were found to cluster according to their respective MLST type with the exception of UW7027-ST78 falling within clade III of ST192 isolates. Branch labels represent a bootstrap with 1000 permutations. Color assignment was done to visually differentiate ST192 clades as defined in this study (green: VRE clade CIa; red: VRE clade CIb; salmon: VRE clade CII; light blue: VSE/VRE clade CIII and grey VSE/VRE clade IV). Outlying brackets frame the year of isolation of the strains belonging to the respective clade. All isolates are ST192 unless stated next to the strain name. Efm_DO represents the reference *E. faecium* DO/TX16 (CP003583). “vanB-“ refers to *vanB*-negative isolates.

Illumina sequencing was carried out for all *E. faecium* ST192 strains to reveal relatedness within and between *vanB*-positive and *vanB*-negative isolates. The available genomic nucleotide sequence of *E. faecium* DO/TX16 served as a reference for subsequent mapping of obtained reads. Due to multiple ambiguities after mapping above a set threshold of 10%, 4 ST192 isolates were omitted from the core genome analyses (Table 1). Called variants were condensed to 8,876 single nucleotide polymorphisms (SNPs), thereby excluding every nucleotide variant which most likely resulted from a recombination event (see material and methods). The inferred maximum likelihood tree groups all but one ST192, UW11625 (*vanA*-positive, but *vanB*-negative), into one of 4 main clades or subclades (Fig. 1a). The proportional transformed branch diagram was chosen, as it explicitly charts the different clades rather than displays the number of sites altered between individual strains (for nucleotide substitutions per site see Supplementary Fig. S1). It is apparent that the German *E. faecium* ST192 population is divided into 2 *vanB*-positive clades or subclades (CIa + b, CII) and 2 *vanB*-negative clades (CIII and CIV), the latter infrequently exhibiting *vanB*-positive and vancomycin-resistant isolates (UW5267, UW8130, UW6376, UW 8173). Clade I is represented by 2 subclades which separates the strains UW8084, UW9284 and UW8260 (CIb) and UW8030 from the bigger branch containing 13 isolates (Fig. 1a). Further, clade II is clearly to be distinguished from CI/III/IV, which is congruent with the strains being isolated from a recent outbreak with *E. faecium* ST192 (*vanB*-positive) on a neonatal care unit^7^. Interestingly, there is no apparent correlation based on the year or the region (federal state) the strains originated from, as for example *vanB*-positive isolates of year 2009 were found both in the *vanB*-positive clade CIa or the “primarily” *vanB*-negative clade CIII (Fig. 1a). Together, the data suggest the dissemination of two main subpopulations of *E. faecium* ST192 in German clinics; one which is represented by the *vanB*-negative and occasionally *vanB*-acquiring clades CIII/CVI; and the second which is composed of an epidemic *vanB*-positive subgroup circulating among hospitals with a geographical preference to the midst and northern parts of Germany (North Rhine-Westphalia, Hamburg, Western Pomerania, Table 1).

A set of non-ST192 were subjected to whole genome sequencing accordingly. Based on 14,177 SNP positions in the core genome, these isolates are only distantly related to the ST192 population (Fig. 1b and Supplementary Fig. S1). Strain *E. faecium* UW7027 (ST78) represents the sole exception as it groups with cluster CIII of *vanB*-negative *E. faecium* ST192 strains (Fig. 1a,b and Supplementary Fig. S1). Further, isolate UW8914 (ST927) is more closely related to the ST192 population of both, *vanB*-positive and *vanB*-negative clades, as is UW11625, an ST192 strain which constitutes an outliner in the ST192 core tree and clusters with an ST203 isolate according to the core genome data (Fig. 1a,b). As demonstrated by confidence intervals, the results obtained underscore the importance of whole genome sequencing (WGS) in analyzing population structure in more depth than conventional multi locus sequence typing of only seven housekeeping genes. However, other sequence types analyzed in this study were either represented by their own clade or as singlets (Fig. 1b and Supplementary Fig. S1).

### Determination of Tn*1549* insertion sites

**Figure 2:**
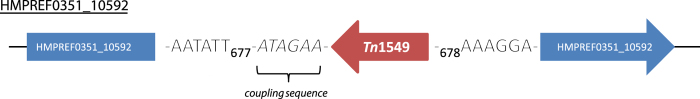
Schematic representation of Tn*1549* insertion into the major insertion site HMPREF0351_10592. Insertion and orientation of Tn*1549* into HMPREF0351_10592 is depicted alongside six nucleotides framing the specific insertion site. Enumeration of nucleotides is according to the coding sequence of HMPREF0351_10592. The coupling sequence represents nucleotides which were co-transferred from the initial donor strain.

Insertion sites of the composite *vanB* transposon Tn*1549* were determined for 38 *vanB*-positive isolates. These included 5 strains which were omitted for core genome analysis (Table 1). In total, 72% of all strains analyzed had the transposon integrated into a novel chromosomal insertion site within the open reading frame HMPREF0351_10592, encoding a hypothetical protein present in *E. faecium* DO only (BlastN search, data not shown) (Table 1). It is worth noting that the three strains UW7606, UW7652 and UW7816 (all ST192) were counted as one in these analyses, as they were part of a suspected outbreak and do not represent independent samples. Except for the three outbreak strains, which exhibit Tn*1549* insertion next to a pathogenicity island (Table 1), all ST192 isolates, as well as the ST78, ST927 and ST202 strains, displayed identical insertion sites with the most frequent locus HMPREF0351_10592. Interestingly, they also comprised the very same coupling sequence of 5 nucleotides (-ATAGAA-) at the 3′- end of Tn*1549* (Fig. 2). Four strains of ST16, ST17 and ST208 showed identical insertion sites into the intergenic region of HMPREF0351_10636 and HMPREF0351_10635. However, the sequence types differed in their coupling sequence and orientation of *Tn*1549 integration (Supplementary Fig. S2). Four isolates of different STs displayed single insertions into genes or intergenic regions widely distributed over the reference genome *E. faecium* DO (Table 1 and Supplementary Fig. S2). We were unable to resolve the insertion site for strain UW8173 (ST192). In summary, all German vancomycin resistant isolates of *E. faecium* analyzed in this study showed novel chromosomal, so far undescribed loci for Tn*1549* integration.

### Phylogeny of Tn*1549*

**Figure 3:**
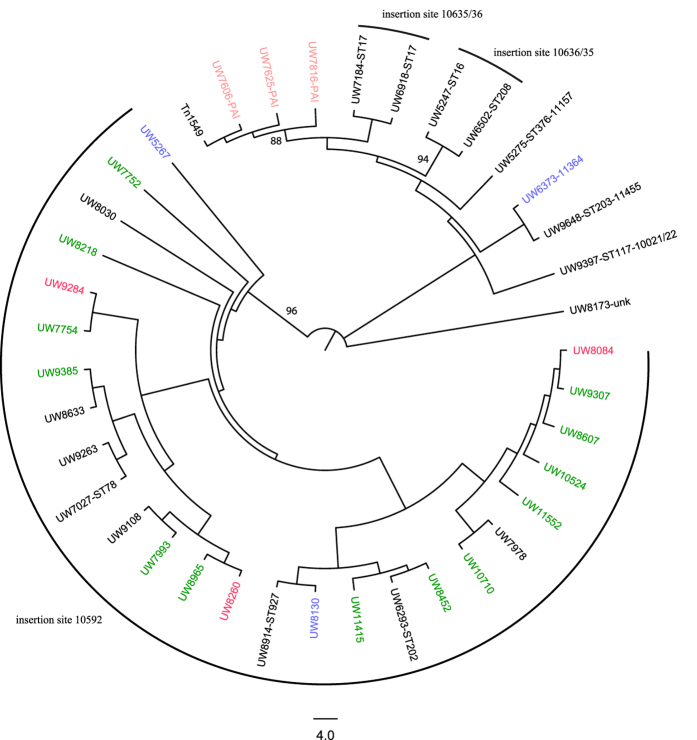
Phylogenetic analysis based on the Tn*1549* sequences of German *E. faecium* ST192 and non-ST192 clinical isolates. The proportional transformed branch diagram revealed an insertion site-specific clustering across all sequence types. Differentiation of the various insertion sites was further validated by bootstrap analysis with 1000 permutations and is indicated by branch labeling. For consistency, color coding represents the different ST192 clades as represented in Fig. 1a,b (green: VRE clade CIa; red: VRE clade CIb; salmon: VRE clade CII; light blue: VSE/VRE clade CIII and grey VSE/VRE clade IV). Unless indicated by specific ST enumeration, all isolates belonged to ST192. Insertion sites are depicted as locus_tag numbering according to the reference genome *E. faecium* DO (CP003583). Tn*1549* represents the reference sequence used for mapping (CP013009). Insertion site “PAI” represents the reference locus_tag EFAU085_02779, as it is not present in *E. faecium* DO, and due to the proximity to a pathogenicity island (PAI) was termed “PAI” in the following. unk insertion site unknown.

In order to examine whether the phylogeny of the core genomes correlates with the relatedness of the *vanB* transposon, reads were mapped to the reference sequence Tn*1549* of strain WCF-TC1 (CP013009) and a maximum likelihood analysis was calculated from 276 SNP positions. Clustering of the Tn*1549* sequences did not match the clades as described for the core genome (Figs 1b and 3). On the contrary, Tn*1549* segments were strictly assorted to independent groups based on their specific site of insertion (Fig. 3). This included strains of different STs which were distantly related according to their core genome data, e.g. UW6293 (ST202) and UW8452 of the ST192 population, but exhibited almost identical *vanB*-containing mobile genetic elements (Figs 1b and 3).

It is worth mentioning that isolate UW8173, located within clade CIII of *vanB*-negative strains, comprised a Tn*1549* element which seemed to be completely different to all other VRE isolates investigated and for which the site of insertion could not be resolved (Figs 1a and 3).

Moreover, the afore-mentioned isolates of ST16, ST17 and ST208 showing identical insertion sites but altered coupling sequences, formed 2 separate clusters in the Tn*1549* analysis (Fig. 3).

Altogether, Tn*1549* elements of German clinical *E. faecium* isolates generally displayed a high degree of identity; however, refined distinction allows clustering of the Tn*1549* sequences based on their site of chromosomal integration, a clustering which is different to their entire core genome background.

### Transfer of Tn*1549* and determination of transferred fragment sizes

The mechanism by which Tn*1549* is transferred from one bacterium to the other is currently under debate as both classical transposition as well as transfer of large chromosomal fragments have been reported to date^10^,^12^,^13^. Our phylogenetic analyses of the transposon and most importantly of the coupling sequences and flanking regions indicate that acquisition of the resistance determinant might rather be due to the transfer of genomic segments than targeted insertion of the sole Tn*1549* element from independent origins. To put the hypothesis to proof, a series of *in vitro* conjugation experiments was conducted with combinations of donor and recipient strains as summarized in Table 2. Generally, Tn*1549* was transferred at variable but low frequencies from 3.3 × 10^−9^ to 1.2 × 10^−6^ transconjugants (TCs) per recipient cells (Table 2). Two *E. faecium* clinical isolates (UW5267, UW8030) failed to transfer Tn*1549* to *E. faecium* 64/3 or 64SS, respectively (Table 2).

We further conducted conjugation experiments using *E. faecalis* OG1RF and JH2-2 as recipient bacteria. No single TC was obtained in filter-mating experiments when *E. faecalis* served as a recipient, even though an appropriate *E. faecium* counterpart such as 64/3 or AK-EM40RF received Tn*1549* and the vancomycin resistance cassette from donors *E. faecium* UW7184 and *E. faecium* UW7606 (Table 2).

A selection of TCs obtained from the 1^st^ round of transfer experiments were subsequently used for further filter-mating analysis. All but one donor TC transferred Tn*1549* to the respective recipients and thus produced a set of 2^nd^ generation and vancomycin-resistant TCs which were included in subsequent analyses of transferred fragment sizes.

In order to determine the size of the transferred elements, one or two TCs per experimental setup were subjected to next generation sequencing. Bioinformatics analyses were based on mapping of contigs obtained by *de novo* assembly to Tn*1549* followed by Mauve alignments of the donor and the respective TC sequences. The accumulation of multiple SNPs in close proximity was defined as the region representing non-transferred DNA and thus set to frame the transferred element. It must be noted that the sizes represent approximations and in some cases could not be resolved in detail due to limited contig lengths. However, as a result, none of the analyzed TCs obtained the sole Tn*1549* transposon by classical transposition, but rather acquired a larger chromosomal fragment of variable size (Table 2). Notably, even 2 TCs isolated from the very same conjugation experiment showed transfer of differently sized genomic fragments. For instance, 7606 × 6711RF × BM4105SS TC1 revealed the acquisition of a >220 kb element whilst the second TC analyzed, 7606 × 6711RF × BM4105SS TC2, received 140 kb only (Table 2).

The MICs to vancomycin of the transconjugants generally resembled those of the donor strains, as for example both above mentioned 2^nd^ generation TCs exhibited the same MIC of 64 μg/ml as their donor 7606 × 6711RF (Table 2).

Furthermore, no association determining the size of the Tn*1549*-containing fragment could be deduced from the combination of donor or recipient strain used. For instance, transconjugant 7184 × 64/3 TC1 acquired a >123 kb fragment, while using the very same strain as a donor in subsequent 2^nd^ rounds of conjugations, the resulting TC (7184 × 64/3 × BM4105SS TC1) obtained 81 kb only (Table 2).

Since Tn*1549* transmission permits carryover of flanking chromosomal regions, TCs which were obtained by using *E. faecium* UW7606 as a donor co-transferred important virulence determinants such as a pathogenicity island alongside the resistance cassette.

Together, our data suggests a Tn*1549*-related transfer different from the supposed mechanism of transposition.

### Characterization of plasmid content of donor, recipient and transconjugant bacteria

The mechanism and putative factors which trigger or support transfer of large Tn*1549*-containing fragments to recipient strains still await elucidation. It was hypothesized by Dahl and colleagues that certain plasmids get transferred simultaneously with Tn*1549* in an undetermined manner^13^, while others did not observe co-transfer of episomal DNA^14^. In order to analyze the putative involvement of a plasmid co-transfer in our conjugation experiments, we first screened a set of donor, recipient and TC strains by PCR using oligonucleotides specifically amplifying replication initiation genes (*rep* genes) of the most abundant enterococcal plasmids as well as the toxin/antitoxin system *axe-txe* of pRUM of *E. faecium* U37 (AF507977.1) or of plasmid pDO2 from *E. faecium* DO (CP003585). Table 3 provides a summary of the *rep*-PCR results, hence indicating the co-transfer of a putative vector carrying *rep*_*17*_*/repA* of pRUM and/or the *axe-txe* system to almost every transconjugant of the 1^st^ and 2^nd^ generation. The only exception was represented by 7606 × AK-EM40RF and 7606 × BM4105RF where either the *rep17*- or the *axe-txe-*specific primers produced a positive PCR product (Table 3).

Sequencing of the *rep* gene of the TCs revealed that it was more related to the *rep* sequence of plasmid 2 of AUS0085 than to that of pRUM. The sequence of the *axe-txe* system of p7606 × 64/3 TC1 (named pWCF-TC1) is identical to both pRUM and plasmid p1 of the Australian isolate AUS0085 (not shown). Thus, these findings suggest the presence of a novel hybrid plasmid containing a *rep17/repA* gene as well as the toxin-antitoxin system or 2 separate plasmids that were co-transferred with Tn*1549*.

**Figure 4:**
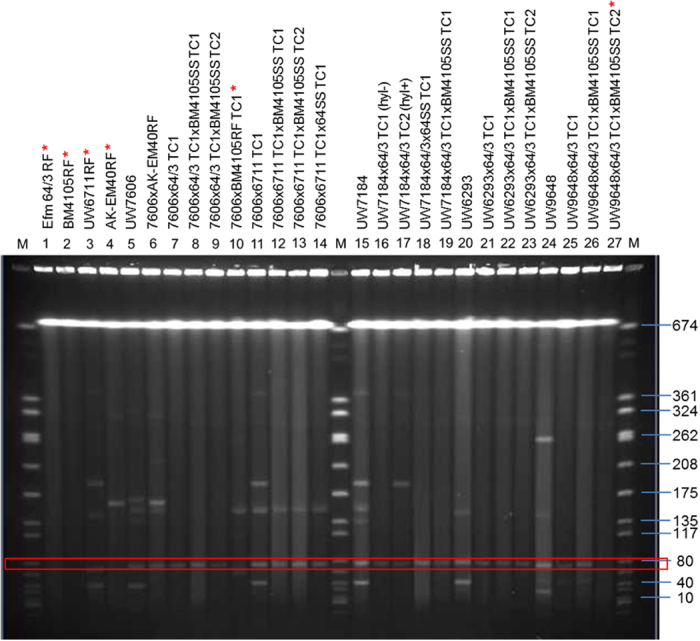
S1-nuclease macrorestriction of donor, recipient and transconjugant (TC) bacteria. Separation in PFGE disclosed the plasmid content of the strains analyzed. The red rectangle indicates the putative novel plasmid pWCF-TC1 of 66.5 kb. The red asterisks indicate strains that do not possess the respective plasmid. M marker strain *S. aureus* NCTC8325.

Enterococcal plasmids are known to exhibit a high degree of modular dissociability^20^. Thus, for in-depth analysis of whole plasmid content, bacterial cells were treated with S1-nuclease and separated by PFGE. As depicted in Fig. 4, donor strains generally contained multiple plasmids. Explicitly prominent, however, was an approx. 40 kb plasmid which was present in almost all German clinical isolates investigated (UW7606, UW6711RF, UW7184, UW6293; Fig. 4). Moreover, a plasmid with the estimated size of 70 kb was observed in all donor strains which produced transconjugants, but absent in the one which failed to transfer Tn*1549* to a receptive host (7606 × BM4105RF TC1; Table 3). This plasmid was the only one which was co-transferred alongside the Tn*1549*-containing chromosomal element.

### Determination of plasmid sequence pWCF-TC1

In this study, we determined the genome sequence of transconjugant *E. faecium* WCF-TC1 (CP013009). PacBio sequencing revealed the presence of one putative plasmid which was co-transferred during filter-mating experiments from the clinical donor isolate *E. faecium* UW7606 to recipient strain *E. faecium* 64/3. The obtained contig was further analyzed to deplete of overlapping sequences and verified for closed circle confirmation by PCR (data not shown). The final plasmid, termed pWCF-TC1, exhibits a length of 66,496 bp and thus is comparable in size to the co-transferred plasmid as estimated by S1-PFGE (Fig. 4). Subsequent annotation by NCBI revealed 76 coding sequences (CDS), amongst them pre-eminently open reading frames for hypothetical proteins, Rep_17_/RepA and the toxin-antitoxin system as described above (CP013010). Interestingly, pWCF-TC1 exhibits a CDS (locus_tag AQ614_13110) encoding a coupling protein of the transfer machinery (TraG), which might represent a supportive factor for Tn*1549* co-transfer. In order to analyze whether the novel plasmid is present in TC-producing strains and TC-non-producers and thus in general might exert co-transfer capabilities on Tn*1549*, sequencing data were mapped to pWCF-TC1 and the amount of ambiguous sites (AS) was used as a rough measure for plasmid/gene existence. Expectantly, strains such as *E. faecium* 64/3 which were plasmid-negative in S1-PFGE analyses displayed no plasmid coverage in mapping attempts (AS 93.7%; Supplementary Table S1). In contrast, 13 of 21 strains analyzed exhibited a substantial coverage with AS in the range of 1.8–7.3%. However, conflicting results were obtained for some isolates which were plasmid-positive (S1-PFGE), but showed poor coverage of the entire novel plasmid sequence, e.g. 7606 × 6711RF × BM4105SS TC1 (AS 94.3%), or did not harbor episomal DNA according to PFGE but displayed a low percentage of ambiguities after mapping analyses (e.g. 7606 × BM4105RF TC1 showing AS of 16.8%; Supplementary Table S1). Further, putative existence of plasmid pWCF-TC1 as inferred from read mappings was not associated with successful transfer of Tn*1549*, as isolates UW8030 or UW8260 did not produce *vanB*-positive TCs but showed a decent coverage of plasmid pWCF1 (AS 7.3% and 2.1%, respectively, Supplementary Table S1).

## Discussion

*E. faecium* ST192 has been recognized as one of three highly prevalent sequence types responsible for causing VRE outbreaks and hospital-associated (HA) bloodstream infections in German hospital patients in recent years^7^. Moreover, emergence of *E. faecium* ST192 as a dominant clone and belonging to a cluster of HA-strains formerly known as clonal complex 17 (CC17), was reported from Sweden and Korea where outbreaks with these strains were noted^21^,^22^,^23^. Interestingly, and opposed to the data of the present study, vancomycin resistance in these strains was mediated by the acquisition of either *vanA*, as part of the plasmid-associated transposon Tn*1546*, or the *vanB*-containing Tn*1549*, also of plasmid origin. Sivertsen and colleagues demonstrated that the *vanB2*-Tn*1549* mobile element inserted into a 40 kb *rep*_17/pRUM_ plasmid which pre-existed in vancomycin-sensitive enterococci (VSE)^22^. Hence, generation of vancomycin-resistant strains was followed by clonal spread of the resistant population. We herein describe the population structure of German VRE ST192 in comparison with further ST lineages, all exhibiting a chromosomally encoded Tn*1549*-*vanB*. However, localization of Tn*1549* in enterococcal chromosomes is no unique feature of German *E. faecium* isolates and, as a matter of fact, had been reported from many countries across the globe^11^,^13^,^24^,^25^.

Based on the core genome data, VRE and VSE of the German ST192 population can be divided into at least two distinct clades; the VRE only clade CIa and the VSE clades CIII/CIV which occasionally acquired the resistance determinant (Fig. 1a). This would suggest the existence of a quite diverse *E. faecium* ST192 population, endemic all across Germany with the capabilities of both, clonal spread of VRE strains as well as *de novo* acquisition of *vanB*-Tn*1549* by susceptible progenitors. In support of this, we recently demonstrated by PFGE and DiversiLab^®^ and by using an expanded set of clinical isolates that German VRE exhibit a versatile population structure across all endemic lineages^26^. Whether the still susceptible population is comprised of pre-existing, HA- and circulating high risk-VSE strains or represent gut commensals highly prevalent in the human community, remains to be determined. This is of major importance in order to implement countermeasures hence preventing transmission of even a certain VSE subpopulation prone to acquire vancomycin resistance.

The hypothesis of *de novo* generation rather than clonal spread of VRE was also presumed by Howden *et al.* in their recent description of 61 *E. faecium* clinical isolates from Australia^19^. The study included 36 VRE strains exhibiting a chromosomally encoded *vanB*-Tn*1549*. The authors compared the transposon sequence, insertion site and orientation of Tn*1549* insertion, as well as the coupling sequences present in clinical *E. faecium* isolates and in anaerobic gut commensals. In accordance with our data, highly similar Tn*1549* were associated with identical coupling sequences and dispersed throughout a phylogenetic tree covering different sequence types^19^. Moreover, Tn*1549* localization revealed seven unique insertion sites. Interestingly, these sites differ from the seven loci for Tn*1549* insertion described in the present study. Also, German and Australian isolates exhibited distinct coupling sequences which provide evidence for independent origins of acquisition and random insertion of Tn*1549* based on classical transposition events at first. Notably, a Tn*1549* sequence obtained from *Clostridium* spp. of another Australian patient was identical to Tn*1549* of a subset of VRE strains, thus indicating that certain gut commensals could act as a reservoir for the *vanB* resistance determinant. Indeed, extensive PCR screenings have previously demonstrated that *vanB* is highly prevalent in non-enterococcal anaerobic bacteria of healthy individuals^16^,^27^. However, other Tn*1549* sequences from anaerobic commensals were shown to be only distantly related to the transposon isolated from VRE strains^19^. This assumes a more frequent transfer among enterococci (VRE and VSE) rather than *de novo* acquisition of Tn*1549* from the gut microbiota.

In accordance with this hypothesis, our study revealed a highly specific clustering of the transposon according to the respective insertion sites (Fig. 3). Due to identical coupling sequences as well as distribution of homologous Tn*1549* elements across the entire phylogenetic tree of the core genome (Fig. 2 and Supplementary Fig. S2), independent acquisition by transposition followed by clonal dissemination of the generated VRE can be excluded. The data are rather indicative of transfer of Tn*1549* based on the mechanism of illegitimate and/or homologous recombination, given the presence of a specific target region in the recipient strain. Consistent with this assumption, filter-mating experiments conducted in this study clearly demonstrated that Tn*1549* exhibits the capability to transfer as part of a large chromosomal fragment. Transfer was also observed to recipient strains which lacked the apparent hot spot for insertion, but subsequently acquired the entire fragment including insertion sites and flanking regions of variable sizes (Tables 1 and 2). However, a certain restriction must be imposed by relatedness of the core genome, especially of the Tn*1549* flanking regions, as no transfer was achieved to the species *E. faecalis*.

It has been reported by a number of studies that the *vanB*-containing mobile genetic element is capable of co-transferring adjacent genomic fragments^10^,^12^,^13^,^14^,^28^,^29^. In one case, concomitant transfer led to the development of strains with increased tolerance to antibiotics^11^. Likewise, we observed co-transfer of a pathogenicity island located in close proximity to the resistance cassette. This is additionally worrisome, as increased prescription and usage of certain antibiotics could trigger the generation and emergence of a bacterial subpopulation with enhanced pathogenicity and/or antibiotic resistances.

It still remains unclear which factors are involved or might trigger the transfer of *vanB*-Tn*1549*, either with or without flanking regions. Our study demonstrated the co-transfer of a novel plasmid, termed pWCF-TC1. Whether it has the capability to mobilize Tn*1549* or is itself dependent on mobilization remains to be determined; however, transfer of vancomycin resistance to recipient bacteria only occurred from donor strains which were shown to be plasmid-positive. Analysis of the gene content revealed the presence of *traG* (locus_tag AQ614_13110) encoding a coupling protein of the transfer machinery. However, presence and/or detailed investigation of the nucleotide sequence of the *traG* open reading frame showed no correlation with the ability of the donor to transfer Tn*1549* and/or the novel plasmid pWCF-TC1 (Supplementary Table S1). As an example, clinical isolate UW8260 was carrying pWCF-TC1 and encodes an intact ORF for *traG* but failed to produce Tn*1549*-*vanB*-positive transconjugants. Collectively, our data suggest the presence and simultaneous transfer of certain plasmids together with the vancomycin resistance transposon Tn*1549*. As enterococcal plasmids are known for their diverse modular structure assignment of transfer capabilities to certain coding sequences cannot be deduced from current data.

In summary, we hypothesize that initial acquisition of Tn*1549* by a receptive *Enterococcus* might occur via transposition from the gut microbiota. However, dissemination of the *vanB* resistance locus in German clinical isolates is presumably due to the exchange of genomic material between VRE and VSE. This might or might not involve the action of co-mobilizing plasmids. It is important to keep in mind the possibility of a circulating high risk VSE lineage in order to prevent *de novo* generation and further spread of dominant VRE HA-populations.

## Methods

### Strain collection

An overview of genotypic and phenotypic features of 49 ST192 and non-ST192 *E. faecium* clinical isolates analyzed in this study and isolated between 2004 and 2014 is given in Table 1. For comparative reasons 11 *vanB*-negative *E. faecium* ST192 strains were included in the strain collection. The strains originated from hospital or diagnostic microbiological laboratories all across Germany and were sent to the National Reference Centre for Staphylococci and Enterococci for resistance determination and further molecular characterization. The majority of these strains were derived from bloodstream infections. Three isolates (UW7606, UW7625, UW7816) belonged to a previously reported outbreak^7^.

### Antimicrobial susceptibility testing

Susceptibility to vancomycin was determined by using the broth microdilution method according to DIN58940 and applying EUCAST breakpoints and, in case of antibiotics with no EUCAST breakpoints, using epidemiological cut-off values (ECOFFs) for interpretation of the results (www.eucast.org). Donor, recipient (Table 1) and transconjugant strains (this study) obtained from filter-mating experiments were analyzed for resistance to vancomycin, fusidic acid, rifampicin, streptomycin and/or spectinomycin, respectively.

### Filter-mating experiments

Transfer capabilities of Tn*1549* were analyzed by filter-mating experiments and by altering donor and recipient strains as stated in Table 2. To discriminate the recipient from the donor strain rifampicin/fusidic acid (RF)- or streptomycin/spectinomycin (SS)-resistant *E. faecium* strains were used (Table 1). Likewise, *E. faecalis* OG1RF or JH2-2 served as recipient bacteria. Putative transconjugants of filter-mating experiments were selected on agar plates containing 5 μg/ml vancomycin, 30 μg/ml rifampicin and 20 μg/ml fusidic acid or 150 μg/ml streptomycin and 150 μg/ml spectinomycin, respectively. Further, transconjugants were routinely screened for acquisition of the *vanB* gene cluster by PCR using oligonucleotides as published previously^7^. Additional evaluation of the transconjugants was carried out by determining susceptibility to vancomycin using broth microdilution assays.

### DNA extraction

Strains were cultivated overnight in brain heart infusion broth at 37 °C. DNA used for PCR or library preparation for Illumina sequencing was extracted by utilizing the DNeasy Blood and Tissue kit according to the manufacturer’s instructions (Qiagen, Hilden, Germany). DNA subjected to sequencing by means of Pacific Bioscience SMRT technology was extracted by using the Genomic-tip 100/G kit (Qiagen, Hilden, Germany).

### Polymerase chain reaction (PCR)

To specifically amplify the ORFs of replicase genes with oligonucleotides rep-p-hylF (5′-TGAGCCCCAAGGGATTCAGGGT-3′) and rep-p-hylR (5′-CGCAATCAGCAAACGGCAAATCG-3′) for pLG1 or primers as published elsewhere^30^,^31^,^32^,^33^, a PCR was carried out as follows: for each sample 12.5 μl of a 2x concentrated DreamTaq Green-Mastermix (Thermo Scientific), as well as forward and reverse primer (final concentration of 0.1 μM) and 0.5 μl of the designated DNA-sample were mixed with DEPC H_2_O to a final volume of 25 μl. Subsequently, DNA amplification was carried out at 95 °C for 120 sec, followed by 30 cycles at 95 °C for 30 sec, annealing temperature of 50 °C (*axe-txe* oligonucleotides only) or 55 °C for 30 sec, elongation at 72 °C for 30 sec and a final extension for 7 min at 72 °C. The same protocol was applied for verification of closed circle conformation of plasmid pWCF-TC1. Oligonucleotides pTC1_cc_fw (5′-CTTAAAGGATGTGTGGATTTAT-3′) and pTC1_cc_rv (5′-CGCTCCGTTTACAGTAATAT-3′) were used at a final concentration of 0.1 μM and an annealing temperature of 55 °C.

### S1-nuclease macrorestriction

Examination of plasmid content of various isolates was conducted by S1-nuclease treatment prior to separation of the genetic content in pulsed-field gel electrophoresis as published previously^32^.

### Single molecule real-time (SMRT) sequencing

A transconjugant strain of *E. faecium* UW7606 (donor) and *E. faecium* 64/3 (recipient), 7606 × 64/3 and termed WCF- TC1 in the following, was sent to GATC (Konstanz, Germany) for whole genome sequencing by means of SMRT technology. The retrieved chromosomal and plasmid contigs were manually trimmed of overlapping sequences utilizing the Geneious software v7.1.4 (Biomatters Ltd.), hence yielding 2 fragments of 2.686.859 bp and 66.496 bp, respectively. Annotation was performed by NCBI.

### Illumina whole genome sequencing and bioinformatic analyses

A total of 1 ng of extracted DNA was used for library generation by utilizing the Nextera XT DNA Library Prep Kit according to the manufacturer’s recommendations (Illumina). Sequencing was carried out on a MiSeq benchtop instrument and performed in paired-end mode using a v3 chemistry-based cartridge 600 (600-Cycle Reagent Kit, Illumina). Obtained reads were mapped to a designated reference sequence by utilizing a pipeline based on BWA version: 0.7.12-r1039 (BWA-SW)^34^ and VarScan v2.3 for variant calling^35^. As enterococci are highly recombinant, description of the core genome requires depletion of SNPs which might result from recombination events. Thus, reads mapped to reference strain *E. faecium* DO/TX16 (CP003583; DO in the following) were subjected to a custom-made script thereby excluding all SNPs falling within 300 bp or less of distance from each other. Retained SNPs served as a basis for phylogenetic analyses by using the graphical user interface of Seaview^36^ in combination with the program PhyML 3.0^37^. Bootstrap confidence intervals are based on 1000 permutations. For visualization purposes, trees were processed with FigTree v1.4.0 (http://tree.bio.ed.ac.uk/software/figtree/) and/or Adobe Illustrator (Adobe Systems). (Sequence read data are available from the SRA database under accession SRP069166).

In order to determine Tn*1549* insertion sites, reads were mapped to the transposon sequence of WCF-TC1 (33.811 bp) (CP013009) and the consensus of read pile ups at both 5′-and 3′-ends of Tn*1549* was extracted to search for homologous regions using BlastN (http://www.ncbi.nlm.nih.gov/). For examination of transferred fragments sizes after conjugation experiments, genomic reads were subjected to *de novo* assembly by utilizing the open source pipeline a5-miseq^38^. The resulting contigs were mapped to Tn*1549* of WCF-TC1 and a consensus sequences including flanking regions was extracted. Subsequently, consensus sequences of transconjugant strains were aligned to the consensus derived from the respective donor strain by using the Mauve plugin in Geneious.

## Additional Information

**Accession codes:** The nucleotide sequences of WCF-TC1 and pWCF-TC1 are available from the GenBank database under the following accession numbers: CP013009; CP013010. 

**How to cite this article**: Bender, J. K. *et al.* Population structure and acquisition of the *vanB* resistance determinant in German clinical isolates of *Enterococcus faecium* ST192. *Sci. Rep.* **6**, 21847; doi: 10.1038/srep21847 (2016).

## Supplementary Material

Supplementary Information

## Figures and Tables

**Table 1:** Strains used in this study.

Strain	ST	Origin/Reference	Year of isolation	MIC VAN [μg/ml]	*vanB*	Insertion site^a^	Ambiguous sites to Efm DO [%]	
*E. faecium* 64/3 and 64SS	ST21	39	−	−	−	−	−	
*E. faecium* AK-EM40RF	ST18	39	−	−	−	−	−	
*E. faecium* BM4105RF and BM4105SS	ST172	39	−	−	−	−	−	
*E. faecium* DO (TX16)	18	40	1998	n.d.	−	−	n.d.	
UW6711RF	192	Berlin	2006	≤1	−	−	−	
*E. faecalis* OG1RF	ST1	41	−	−	−	−	−	
*E. faecalis* JH2-2	ST8	42	−	−	−	−	−	
*E. faecium ST192 clinical isolates*								
UW5267	192	North Rhine-Westphalia	2004	32	+	10592	6,2	
UW5909	192	Baden-Wuerttemberg	2004	16	−	−	7,2	
UW6373	192	Bavaria	2005	16	+	11364	9,5	
UW6715	192	Berlin	2006	≤1	−	−	8,7	
UW6954	192	Schleswig-Holstein	2006	≤1	−	−	6,9	
UW6982	192	Thuringia	2006	≤1	−	−	6,7	
UW7117	192	Lower Saxony	2007	≤1	−	−	8,6	
UW7162	192	Lower Saxony	2007	≤1	−	−	7,8	
UW7373	192	Bavaria	2008	≤1	−	−	7,3	
UW7606	192	Bavaria	2008	32	+	PAI^b^	8,3	
UW7625	192	Bavaria	2008	2	+	PAI	8,6	
UW7752	192	North Rhine-Westphalia	2009	16	+	10592	7,2	
UW7754	192	North Rhine-Westphalia	2009	32	+	10592	8,5	
UW7816	192	Bavaria	2008	2	+	PAI	8,2	
UW7993	192	North Rhine-Westphalia	2009	64	+	10592	7,7	
UW8030	192	Hesse	2009	64	+	10592	8,3	
UW8080-1	192	Saxony-Anhalt	2009	≤1	−	−	8,6	
UW8084	192	Bavaria	2009	32	+	10592	7,7	
UW8130	192	Bavaria	2010	128	+	10592	7,9	
UW8161-1	192	Saxony-Anhalt	2010	≤1	−	−	7,1	
UW8173	192	Schleswig-Holstein	2010	64	+	unknown	9,5	
UW8218	192	North Rhine-Westphalia	2010	32	+	10592	8,0	
UW8260	192	Saxony	2010	256	+	10592	7,9	
UW8452	192	Hesse	2010	32	+	10592	7,6	
UW8578	192	Bavaria	2011	≤1	−	−	7,9	
UW8607	192	North Rhine-Westphalia	2011	32	+	10592	8,0	
UW8965	192	Rhineland-Palatinate	2011	32	+	10592	8,4	
UW9284	192	Berlin	2011	32	+	10592	8,0	
UW9307	192	Hesse	2011	64	+	10592	8,6	
UW9385	192	Bavaria	2011	32	+	10592	7,4	
UW10524	192	Mecklenburg-Western Pomerania	2013	128	+	10592	7,0	
UW10710	192	Mecklenburg-Western Pomerania	2013	512	+	10592	6,8	
UW11415	192	North Rhine-Westphalia	2013	32	+	10592	6,8	
UW11552	192	Lower Saxony	2014	64	+	10592	7,0	
UW11625	192	North Rhine-Westphalia	2014	512	−	−	8,2	
*Non-ST192 E. faecium clinical isolates*								
UW5247	16	Baden-Wuerttemberg	2004	8	+	10635/10636	8,3	
UW6918	17	Berlin	2007	8	+	10636/10635	5,1	
UW7184	17	Berlin	2007	16	+	10636/10635	6,7	
UW7027	78	Schleswig-Holstein	2007	32	+	10592	6,8	
UW9397	117	Berlin	2012	2	+	10021/10022	6,8	
UW6293	202	Hesse	2005	128	+	10592	5,0	
UW9648	203	Bavaria	2012	128	+	11455	7,7	
UW5275	376	Lower Saxony	2004	8	+	11157	6,2	
UW8914	927	North Rhine-Westphalia	2011	64	+	10592	9,7	
*Strains omitted in core genome analysis, but included in characterization of Tn1549*								
UW7978	ST192	Saxony	2009	32	+	10592	16	
UW8633	ST192	North Rhine-Westphalia	2011	32	+	10592	12,1	
UW9108	ST192	Lower Saxony	2011	32	+	10592	10,2	
UW9263	ST192	Lower Saxony	2011	32	+	10592	11,2	
UW6502	ST208	Bavaria	2006	4	+	10635/10636	13,3	

^a^Insertion sites are depicted as locus_tag numbers according to the reference genome *E. faecium* DO.

^b^“PAI” refers to Tn*1549* insertion in close proximity to a pathogenicity island.

**Table 2:** Overview and results of *in vitro* filter-mating experiments.

Donor	Recipient	Identifier of analyzed transconjugant (TC)	Transfer efficiency^b^	Size of transferred *Tn*1549 fragment [kb]	VAN MIC [μg/ml]^e^
UW5267	64/3	−	−	−	−
UW6293	64/3	6293 × 64/3 TC1	3.3 × 10^−9^	>173^c^	128
6293 × 64/3 TC1	BM4105SS	6293 × 64/3 × BM4105SS TC1 + TC2	5.2 × 10^−8^	50 + 246	256 + 256
UW7184	64/3	7184 × 64/3 TC1	1.4 × 10^−8^	>123^c^	32
7184 × 64/3 TC1	64SS	7184 × 64/3 × 64SS TC1	3.3 × 10^−7^	−d	32
7184 × 64/3 TC1	BM4105SS	7184 × 64/3 × BM4105SS TC1	1.0 × 10^−7^	81	32
UW7184	OG1RF	−	−	−	−
UW7184	JH2-2	−	−	−	−
UW7606	64/3	7606 × 64/3 TC1(WCF-TC1)^7^ ^a^	1.7 × 10^−8^	600	64
UW7606	OG1RF	−	−	−	−
UW7606	JH2-2	−	−	−	−
UW7606	AK-EM40RF	7606xAK-EM40RF TC1	2 × 10^−8^	110	8
UW7606	BM4105RF	7606 × BM4105RF TC1	n.d.	>230^c^	8 (after 48h)
UW7606	UW6711RF	7606 × 6711RF TC1	9.6 × 10^−8^	>214^c^	64
7606 × 64/3 TC1 (WCF-TC1)	BM4105SS	7606 × 64/3 × BM4105SS TC1 + TC2	5.9 × 10^−8^	>220^c^ + 140	32 + 32
7606 × 6711RF TC1	BM4105SS	7606 × 6711RF × BM4105SS TC1 + TC2	3.9 × 10^−8^	213 + 214	64 + 64
7606 × 6711RF TC1	64SS	7606 × 6711RF × 64SS TC1	7.6 × 10^−9^	>214^c^	64
7606 × BM4105RF TC1	64SS	−	−	−	−
UW8030	64/3	−	−	−	−
UW9648	64/3	9648 × 64/3 TC1	8.9 × 10^−9^	>98^c^	64
9648 × 64/3 TC1	BM4105SS	9648 × 64/3 × BM4105SS TC1	1.2 × 10^−6^	>160^c^	64

^a^The nucleotide sequences of the genomic content was determined and deposited at GenBank under accession numbers CP013009, CP013010.

^b^Transfer efficiency refers to the number of transconjugants per recipient cell.

^c^The exact fragment size could not be determined due to limited contig length.

^d^The fragment size could not be determined as donor and recipient strain share the same genomic background.

^e^The minimal inhibitory concentration was routinely determined for all TCs only once. n.d. not determined.

**Table 3:** Overview and results of the PCR analyzing *rep* genes and the *axe-txe* system of prevalent enterococcal plasmids.

Strain	pIP501	pLG1	pVEF	pRE25	pRUM	*axe-txe*
64/3	−	−	−	−	−	−
64SS	−	−	−	−	−	−
BM4105RF	−	−	−	−	−	−
BM4105SS	−	−	−	−	−	−
UW6711RF	−	+	+	−	+	+
AK-EM40RF	−	+	+	−	−	−
**UW7606**	−	+	+	−	+	+
7606 × AK-EM40RF	−	+	+	−	−	+
7606 × 64/3 TC1 (WCF-TC1)	−	−	−	−	+	+
7606 × 64/3 TC1 × BM4105SS TC1	−	−	−	−	+	+
7606 × 64/3 TC1 × BM4105SS TC2	−	−	−	−	+	+
7606 × BM4105RF TC1	−	−	−	−	+	−
7606 × 6711 TC1	−	+	+	−	+	+
7606 × 6711 TC1 × BM4105SS TC1	−	−	−	−	+	+
7606 × 6711 TC1 × BM4105SS TC2	−	−	−	−	+	+
7606 × 6711 TC1 × 64SS TC1	−	−	−	−	+	+
**UW7184**	−	+	+	−	+	+
7184 × 64/3 TC1	−	−	−	−	+	+
7184 × 64/3 × 64SS TC1	−	−	−	−	+	+
7184 × 64/3 TC1 × BM4105SS TC1	−	−	−	−	+	+
**UW6293**	−	+	+	−	+	+
6293 × 64/3 TC1	−	−	−	−	+	+
6293 × 64/3 TC1 × BM4105SS TC1	−	−	−	−	+	+
6293 × 64/3 TC1 × BM4105SS TC2	−	−	−	−	+	+
**UW9648**	−	+	+	−	+	+
9648 × 64/3 TC1	−	−	−	−	+	+
9648 × 64/3 TC1 × BM4105SS TC1	−	−	−	−	+	+

“+”/“−” indicate a positive or negative PCR result, respectively.
