# Supplementary material for: Population structure and acquisition of the vanB resistance determinant in German clinical isolates of Enterococcus faecium ST192
Source: Sci Rep. 2016 Feb 23;6:21847. doi: 10.1038/srep21847 (PMC4763178; doi:10.1038/srep21847)

Population structure and acquisition of the *vanB* resistance determinant in German clinical isolates of *Enterococcus faecium* ST192

Jennifer K. Bender, Alexander Kalmbach, Carola Fleige, Ingo Klare, Stephan Fuchs, Guido Werner

**Supplementary information**

**Fig S1. Phylogenetic analysis of the core genome of German *E. faecium* ST192 (a) and non-ST192 (b) clinical isolates.**

(a) Maximum likelihood (ML) analyses revealed 5 different clades of ST192 isolates. (b) Due to ML analyses, ST192 and non-ST192 were found to cluster according to their respective MLST type with the exception of UW7027-ST78 falling within clade III of ST192 isolates. Branch labels represent a bootstrap with 1000 permutations. Color assignment was done to visually differentiate ST192 clades as defined in this study (green: VRE clade CIa; red: VRE clade CIb; salmon: VRE clade CII; light blue: VSE/VRE clade CIII and grey VSE/VRE clade IV). Outlying brackets frame the year of isolation of the strains belonging to the respective clade. All isolates are ST192 unless stated next to the strain name. Efm_DO represents the reference *E. faecium* DO/TX16 (CP003583). “vanB-“ refers to *vanB*-negative isolates.

**Fig S2. Schematic representation of Tn*1549* insertion into different insertion sites.** Insertion and orientation of Tn*1549* insertion is depicted alongside six nucleotides framing the specific insertion site (A-G). Blue arrows indicate genes affected by or framing Tn*1549* insertion and are named according to the locus_tag of the reference genomes *E. faecium* DO or *E. faecium* AUS0085. If applicable, enumeration of nucleotides follows the coding sequence of the reference genes. In case of intergenic insertion of Tn*1549* nucleotide enumeration was omitted. The coupling sequence represents nucleotides which were co-transferred from the initial donor strain. Please note that a 5-nucleotide base pair difference was detected in all isolates with insertion site “PAI” when compared to the Australian reference genome of *E. faecium* AUS0085 (G).

**Fig S3. Phylogenetic analysis of the Tn*1549* sequence of German *E. faecium* ST192 and non-ST192 clinical isolates.**

Maximum likelihood analyses revealed an insertion site-specific clustering across all sequence types. Differentiation of the various insertion sites was further validated by bootstrap analysis with 1000 permutations and is indicated by branch labeling. For consistency, color coding represents the different ST192 clades as represented in Fig.1a+b (green: VRE clade CIa; red: VRE clade CIb; salmon: VRE clade CII; light blue: VSE/VRE clade CIII and grey VSE/VRE clade IV). Unless indicated by specific ST enumeration, all isolates belonged to ST192. Insertion sites are depicted as locus_tag numbering according to the reference genome *E. faecium* DO (CP003583). Tn*1549* represent the reference sequence used for mapping. Insertion site “PAI” represents the reference locus_tag EFAU085_02779, as it is not present in *E. faecium* DO, and due to the proximity to a pathogenicity island (PAI) was termed “PAI” in the following. unk insertion site unknown.

**Table S1. Mapping of Illumina reads to reference plasmid pWCF-TC1.**

| **Strain** | **putative pWCF1**  **(S1-PFGE)[Y/N]** | **Ambiguous sites [%] after mapping to plasmid pWCF1** | **presence of *traG* [Y/N]†** | **TC produced [Y/N]** |
| --- | --- | --- | --- | --- |
| 64/3 | N | **93.7** | N | n.a. |
| BM4105RF | N | **97.4** | N | n.a. |
| UW6293 | Y | 3.2 | Y | Y |
| UW6711RF | N | **13.6** | Y | n.a. |
| UW7606 | Y | 2.8 | Y | Y |
| UW7184 | Y | 4.2 | Y | Y |
| UW8030 | n.d. | 7.3 | Y | **N** |
| UW8260 | n.d. | 2.1 | Y | **N** |
| UW9648 | Y | 6 | Y | Y |
| 6293x64/3 TC1 | Y | 1.8 | Y | Y |
| 6293x64/3xBM4105SS TC1 | Y | 1.9 | Y | n.a. |
| 7606x64/3 TC1 (WCF-TC1) | Y | 2.8 | Y | Y |
| 7606x64/3xBM4105SS TC1 | Y | **60.3** | Y | n.a. |
| 7606x6711RF TC1 | Y | 2.4 | Y | Y |
| 7606x6711RFx64SS TC1 | Y | 1.9 | Y | n.a. |
| 7606x6711RFxBM4105SS TC1 | Y | **94.3** | N | n.a. |
| 7606xBM4105RF TC1 | N | **16.8** | Y | n.a. |
| 7184x64/3 TC1 | Y | 3.4 | Y | Y |
| 7184x64/3x64SS TC1 | Y | 5.3 | Y | n.a. |
| 9648x64/3 TC1 | Y | **60.9** | Y | Y |
| 9648x64/3xBM4105SS TC1 | Y | **97.4** | Y | n.a. |
| †presence (Y) was confirmed after *de novo* assembly and a minimal nucleotide sequence identity of 70% to *traG* of pWCF1; TC transconjugant; n.a. not analyzed; n.d. not determined | | | | |


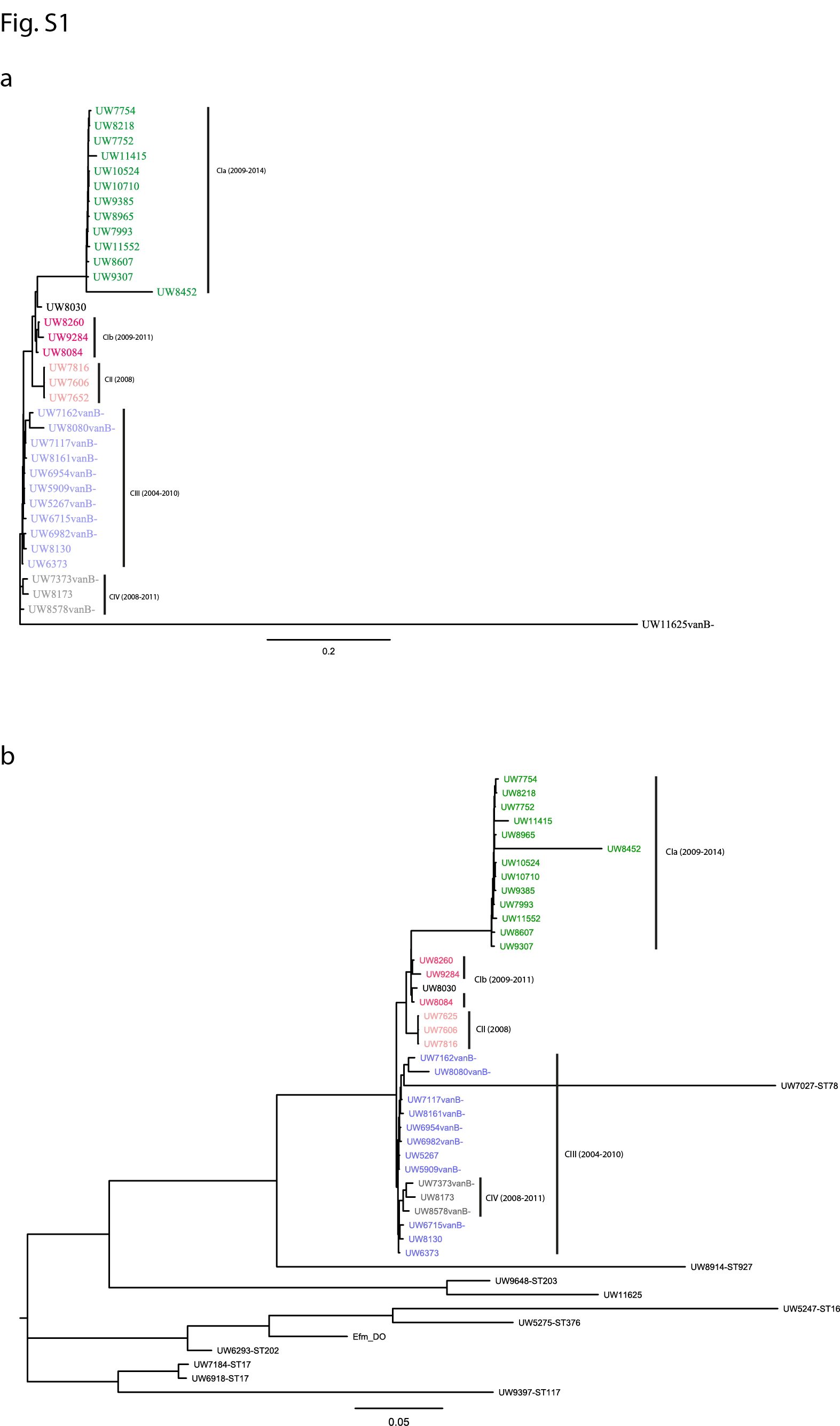


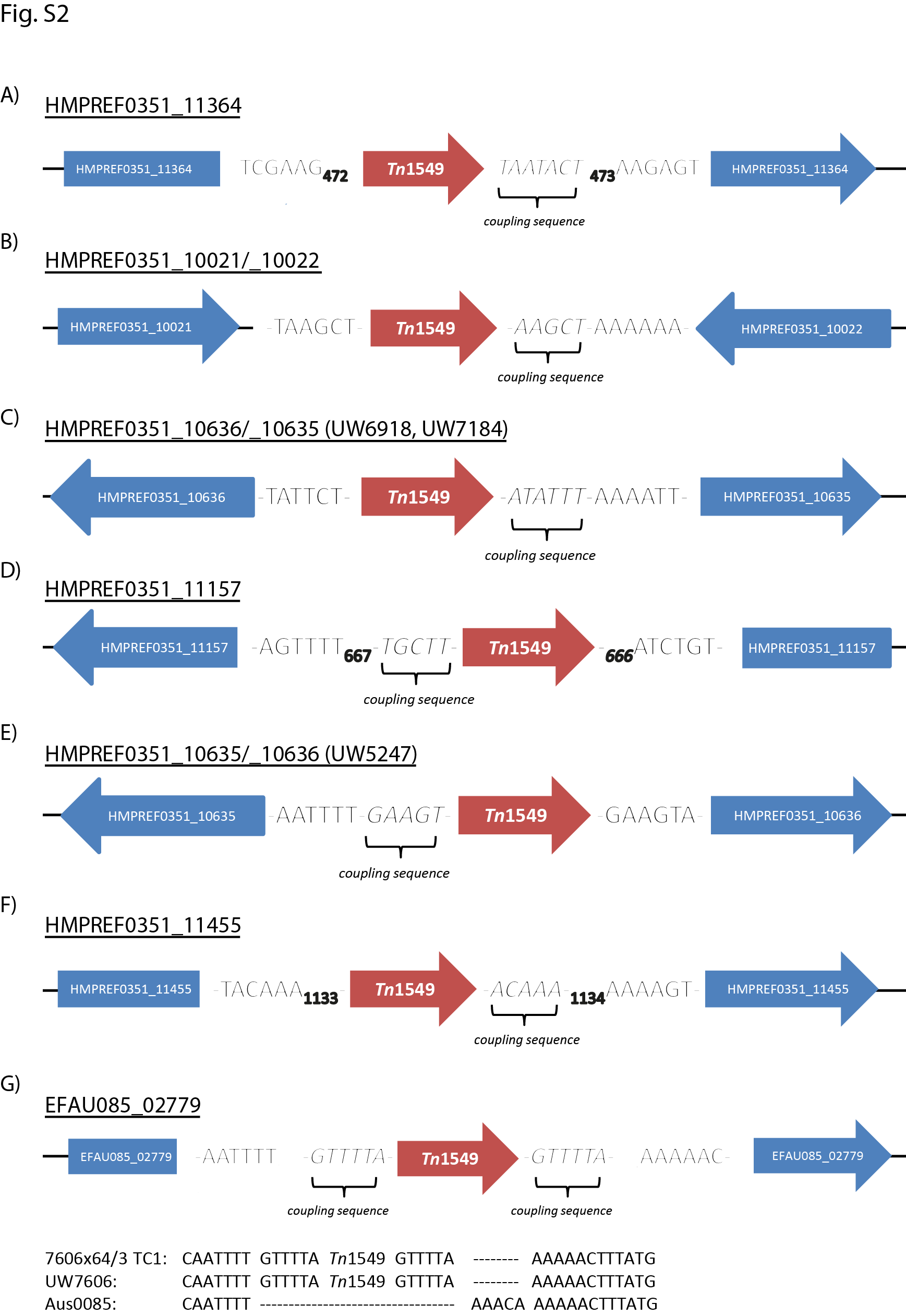


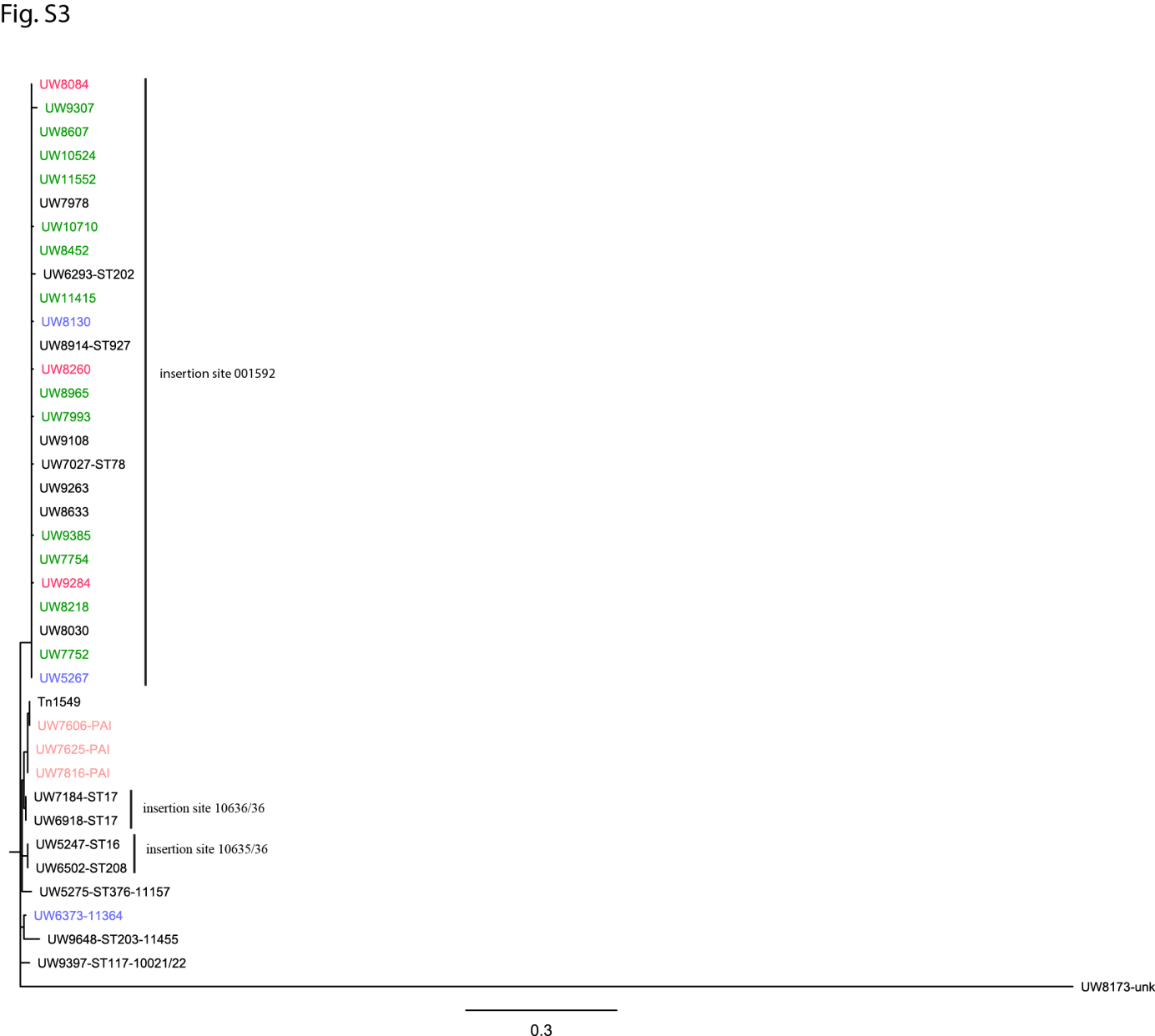

Supplement: Supplementary Information [file srep21847-s1.docx]
